# Cumulative Live Birth Rates of Good and Low Prognosis Patients According to POSEIDON Criteria: A Single Center Analysis of 18,455 Treatment Cycles

**DOI:** 10.3389/fendo.2019.00409

**Published:** 2019-06-26

**Authors:** Wenhao Shi, Hanying Zhou, Li Tian, Zhenghao Zhao, Wei Zhang, Juanzi Shi

**Affiliations:** ^1^The Assisted Reproduction Center, Northwest Women's and Children's Hospital, Xi'an, China; ^2^Department of Respiratory Medicine, Shaanxi Provincial People's Hospital, Xi'an, China

**Keywords:** POSEIDON, cumulative live birth, implantation rate, miscarriage rate, low prognosis patient

## Abstract

**Objective:** To investigate the characteristics and outcomes of low prognosis patients defined by POSEIDON criteria undergoing IVF treatment.

**Design:** Retrospective cohort analysis.

**Setting:** An IVF clinic in a public hospital.

**Patients:** 18,455 fresh aspirated IVF cycles with subsequently frozen embryo transfer from Jan 2014 to Jan 2017 in a single IVF clinic were included in the analysis. The low prognosis patients were categorized into 4 groups based on POSEIDON criteria: group 1: age < 35, antral follicle count (AFC) ≥ 5, number of oocytes retrieved ≤ 9 in the previous cycle; group 2: age ≥ 35, AFC≥5, number of oocytes retrieved ≤ 9 in the previous cycle; group 3: age < 35, AFC < 5; group 4: age ≥ 35, AFC < 5. The non-low prognosis patients: group 5: AFC ≥ 5, previous number of oocytes retrieved > 9 oocytes; group 6: AFC ≥ 5, no previous ovarian stimulation.

**Intervention(s):** None.

**Main Outcome Measure:** The primary outcome was cumulative live birth rate (CLBR).

**Result(s):** Taking group 1 as reference, the CLBR from young women in group 3 (35.5%, OR 0.9, 95% CI 0.7–1.2) was slightly lower than that in group 1 (44.6%, *p* = 0.615). The CLBR in group 2 (24.5%, OR 0.6, 95% CI 0.4–0.8, *p* = 0.004) and group 4 (12.7%, OR 0.4, 95% CI 0.3–0.6, *p* < 0.001) was significant lower than that in group 1. In non-poor prognosis patients, the CLBR from young women in group 5 (53.5% OR 1.3 95% CI 0.9, 1.7, *p* = 0.111) was a slight higher than the reference group 1 while the highest CLBR was originated from the first IVF patients with good ovarian reserve in group 6 (66.9%, OR 2.0, 95% CI 1.6, 2.4).

**Conclusion(s):** The CLBRs and implantation rates in the young women (group 3) with diminished ovarian reserve was similar in those young women (group 1), and was significantly higher than in advanced age women with a fair ovarian reserve (group 2). Though patients in group 2 had better ovarian reserve, more oocytes and more embryos, the pregnancy outcome was inferior to that of group 3 patients with poorer ovarian reserve, fewer oocytes and fewer embryos.

## Introduction

Recently a novel system, the POSEIDON criteria, was developed to classify infertility patients with low prognosis undergoing assisted reproductive technology (ART) treatment (1). It is a useful system for the identification and classification of patients with impaired ovarian reserve or poor ovarian response (POR), providing guidance for the diagnosis and management of these patients (2). Four subsets have been suggested based on quantitative and qualitative parameters including, age, ovarian reserve biomarkers, and ovarian response. The new criteria, by introducing a more detailed stratification of POR, significantly reduced the heterogeneity of patients meeting the Bologna criteria (3), which may differentiate patient subsets within the POR population who could be identified and benefit from specific interventions (4). Although the POSEIDON criteria were established, along with some specific treatment recommendations proposed for the specific patient subgroups (5), there still remains insufficient evidence to support the validity of parameters used in the POSEIDON criteria, as well as the outcome assessment of different subgroups.

Among the four groups based on the POSEIDON criteria, group 1 is undoubtedly the best prognostic group considering their younger age and normal ovarian reserve, while group 4 has the worst prognosis due to the advanced age and diminished ovarian reserve. However, an interesting question is who would achieve better pregnancy outcome, the older women (group 2) with normal ovarian reserve or the young women (group 3) with diminished ovarian reserve. The cumulative live birth rate (CLBR) is considered a preferable measure of success of IVF treatment (6). Until now, there have been very few reports on the CLBRs of the four patient groups defined by the POSEIDON criteria.

The characteristics and prognosis of patients should be used to develop clinical management strategies. The objective of this study is to characterize the low prognosis patients in order to facilitate treatment decision making. In this study, the baseline characteristics and outcomes of patient groups defined by the POSEIDON criteria were analyzed, and CLBR resulting from one aspirated *in-vitro* fertilization (IVF)/intracytoplasmic sperm injection (ICSI) cycle was proposed as the primary outcome measurement for low prognosis patients undergoing IVF treatment (7).

## Materials and Methods

This retrospective study included 18,455 fresh aspirated IVF cycles with subsequent frozen embryo transfers from January 2014 to January 2017 in our center. The live birth outcome was followed up for at least 2 years until Jan 2019. The study was approved by the Ethics Committee for the Clinical Application of Human Assisted Reproductive Technology of Northwest Women's and Children's Hospital (No. 2018002). The ethics committee approved this study waived the need to obtain informed consent. All research was performed in accordance with relevant guidelines and regulations.

### Inclusion Criteria

All fresh IVF/ICSI cycles and subsequently frozen embryo transfers from oocyte retrievals performed in our clinic from January 2014 to January 2017 were included in the analysis. The following cycles were excluded: (1) donated oocyte cycles (*n* = 28), oocyte freezing cycles (*n* = 8); (2) PGS/PGD cycles (*n* = 132); (3) cycles without live birth but with extra frozen embryos during this period (*n* = 337); (4) cycles of patients lost to follow-up (*n* = 41); (5) cycles with induced abortion (*n* = 18).

Patients were categorized according to POSEIDON criteria:

Low prognosis patients
Group 1 (*n* = 879 cycles): Age < 35, antral follicle count (AFC) ≥ 5, number of oocytes retrieved ≤ 9 in the previous cycle;
Group 2 (*n* = 482 cycles): Age ≥ 35, AFC≥5, previous number of oocytes retrieved ≤ 9 in the previous cycle;
Group 3 (*n* = 858 cycles): Age < 35, AFC < 5;
Group 4 (*n* = 1,306 cycles): Age ≥ 35, AFC < 5;
Non-low prognosis patients
Group 5 (*n* = 664 cycles): AFC ≥ 5, previous ovarian stimulation > 9 oocytes;
Group 6 (*n* = 13708 cycles): AFC ≥ 5, no previous ovarian stimulation.

**Figure 1:**
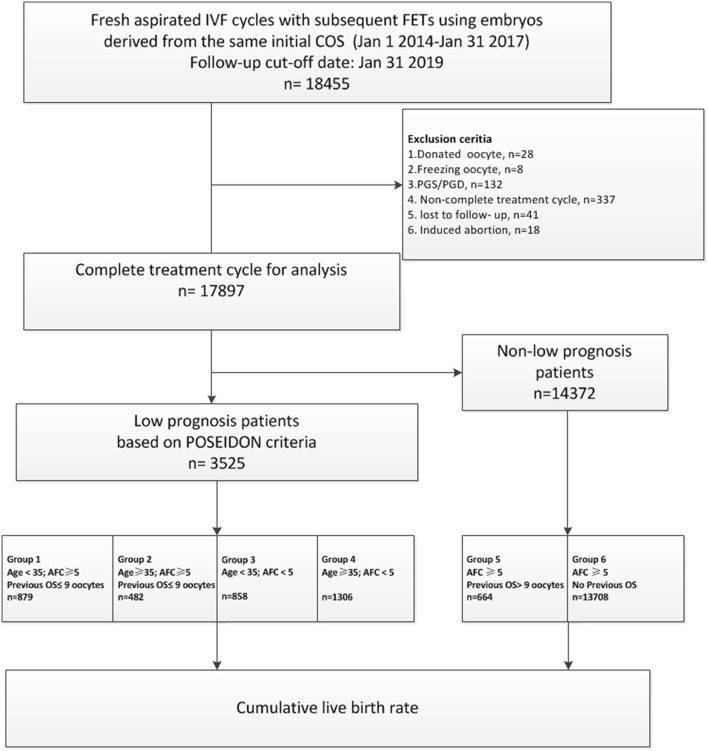
Flow chart and data processing.

Flow chart and data processing procedure are listed in Figure 1. Demographics and basal characteristics of patients are presented in Table 1.

**Table 1:** Demographics and baseline characteristics.

**Group**	**POSEIDON group**				**Non-POSEIDON group**		***P*-value**
	**1**	**2**	**3**	**4**	**5**	**6**	
*N*	879	482	858	1306	664	13708	
**Year of treatment**							<0.001
2014	114 (13.0%)	79 (16.4%)	184 (21.4%)	285 (21.8%)	96 (14.5%)	4154 (30.3%)	
2015	296 (33.7%)	150 (31.1%)	227 (26.5%)	344 (26.3%)	200 (30.1%)	4251 (31.0%)	
2016-2017.01	469 (53.4%)	253 (52.5%)	447 (52.1%)	677 (51.8%)	368 (55.4%)	5303 (38.7%)	
Age of female	29.9 ± 2.8	39.1 ± 3.3	30.2 ± 2.9	40.5 ± 3.5	30.9 ± 4.6	29.9 ± 4.3	<0.001
**Age of female**							<0.001
≤ 30	487 (55.4%)	0 (0.0%)	438 (51.0%)	0 (0.0%)	356 (53.6%)	8375 (61.1%)	
>30, ≤ 35	392 (44.6%)	63 (13.1%)	420 (49.0%)	104 (8.0%)	202 (30.4%)	3823 (27.9%)	
>35, ≤ 40	0 (0.0%)	262 (54.4%)	0 (0.0%)	594 (45.5%)	85 (12.8%)	1224 (8.9%)	
>40	0 (0.0%)	157 (32.6%)	0 (0.0%)	608 (46.6%)	21 (3.2%)	286 (2.1%)	
**BMI of female**							<0.001
≥24	228 (26.1%)	124 (26.2%)	217 (25.6%)	416 (32.3%)	180 (27.3%)	3622 (26.7%)	
≥18.5, <24	529 (60.6%)	315 (66.6%)	560 (66.0%)	813 (63.2%)	422 (64.0%)	8705 (64.1%)	
<18.5	116 (13.3%)	34 (7.2%)	72 (8.5%)	57 (4.4%)	57 (8.6%)	1248 (9.2%)	
Basal FSH (IU/ml)	7.4 ± 2.7	8.4 ± 3.5	9.3 ± 5.5	11.1 ± 7.0	6.6 ± 2.0	6.8 ± 2.6	<0.001
**Type of infertility**							<0.001
Primary	526 (59.8%)	123 (25.5%)	513 (59.8%)	274 (21.0%)	356 (53.6%)	7979 (58.2%)	
Secondary	353 (40.2%)	359 (74.5%)	345 (40.2%)	1032 (79.0%)	308 (46.4%)	5729 (41.8%)	
**Length of infertility, year**							<0.001
≤ 2	306 (34.9%)	193 (40.3%)	309 (36.3%)	509 (39.7%)	255 (38.6%)	5267 (38.9%)	
>2, ≤ 5	391 (44.6%)	134 (28.0%)	394 (46.2%)	319 (24.9%)	264 (39.9%)	5899 (43.5%)	
>5	179 (20.4%)	152 (31.7%)	149 (17.5%)	454 (35.4%)	142 (21.5%)	2385 (17.6%)	
**AFC**							<0.001
<4	0 (0.0%)	0 (0.0%)	521 (60.7%)	885 (67.8%)	0 (0.0%)	0 (0.0%)	
≥4, <10	484 (55.1%)	380 (78.8%)	337 (39.3%)	421 (32.2%)	148 (22.3%)	3844 (28.0%)	
≥10	395 (44.9%)	102 (21.2%)	0 (0.0%)	0 (0.0%)	516 (77.7%)	9864 (72.0%)	
**Main etiology**							<0.001
Pelvic-tubal factor	588 (67.3%)	342 (71.4%)	536 (62.9%)	761 (59.3%)	445 (67.5%)	8809 (64.9%)	
Ovarian factor	73 (8.4%)	36 (7.5%)	169 (19.8%)	283 (22.1%)	48 (7.3%)	1156 (8.5%)	
Male factor	94 (10.8%)	38 (7.9%)	36 (4.2%)	39 (3.0%)	82 (12.4%)	1747 (12.9%)	
Endometriosis	27 (3.1%)	2 (0.4%)	49 (5.8%)	33 (2.6%)	7 (1.1%)	198 (1.5%)	
Uterine factor	10 (1.1%)	17 (3.5%)	22 (2.6%)	83 (6.5%)	6 (0.9%)	223 (1.6%)	
Other reasons	82 (9.4%)	44 (9.2%)	40 (4.7%)	84 (6.5%)	71 (10.8%)	1438 (10.6%)	
**Female smoking**							0.509
No	2 (0.2%)	0 (0.0%)	2 (0.2%)	0 (0.0%)	1 (0.2%)	15 (0.1%)	
Yes	877 (99.8%)	482 (100.0%)	856 (99.8%)	1306 (100.0%)	663 (99.8%)	13693 (99.9%)	
**Gravidity**							<0.001
0	514 (58.7%)	116 (24.3%)	506 (59.2%)	248 (19.2%)	356 (53.8%)	7895 (57.7%)	
1	214 (24.4%)	132 (27.6%)	196 (22.9%)	306 (23.7%)	166 (25.1%)	3048 (22.3%)	
≥2	148 (16.9%)	230 (48.1%)	153 (17.9%)	737 (57.1%)	140 (21.1%)	2738 (20.0%)	
**Parity**							<0.001
0	820 (93.6%)	293 (61.2%)	790 (92.3%)	646 (50.0%)	588 (88.8%)	12020 (87.8%)	
1	54 (6.2%)	158 (33.0%)	65 (7.6%)	560 (43.3%)	63 (9.5%)	1511 (11.0%)	
≥2	2 (0.2%)	28 (5.8%)	1 (0.1%)	86 (6.7%)	11 (1.7%)	157 (1.1%)	
**Number of oocytes retrieved in the previous cycle**							NA
>10	0 (0.0%)	0 (0.0%)	4 (1.7%)	6 (1.1%)	559 (84.2%)	0 (0.0%)	
>4, ≤ 10	539 (61.3%)	210 (43.6%)	30 (12.4%)	57 (10.6%)	105 (15.8%)	0 (0.0%)	
≤ 4	340 (38.7%)	272 (56.4%)	208 (86.0%)	473 (88.2%)	0 (0.0%)	0 (0.0%)	

*AFC, antral follicle count; FSH, follicle stimulating hormone; OS, ovarian stimulation*.

*Mean + SD / N (%), calculated using EmpowerStats (www.empowerstats.com) and R*.

*Kruskal Wallis Rank Test continuous variables, Chi-square tests for categorical variables, Fisher Exact for categorical variables with Expects <10*.

### Ovarian Stimulation and Oocyte Retrieval

The protocol for ovarian stimulation (OS) was determined individually according to female age, body mass index (BMI), basal follicle stimulating hormone (FSH) and antral follicle count (AFC). 94.33% of IVF patients received recombinant and/or urinary gonadotrophins (rFSH/hMG) in GnRH agonist protocol or GnRH antagonist protocol followed by IVF or ICSI. For women with diminished ovarian reserve, the mild stimulation protocol or luteal phase ovarian stimulation or natural cycle was used. Human menopausal gonadotrophin (hMG, Li Zhu, China) was added in mild ovulation protocol or Shanghai protocol according to patients' response to stimulation. Human chorionic gonadotrophin (hCG) 4,000–10,000 IU or recombinant hCG (r-hCG, MerckSerono S.p.A.) 250μg was administered when 2-3 follicles reached the size of 17 mm or higher. Thirty-six hours later, oocyte retrieval was performed using transvaginal ultrasonography-guided aspiration. The ovarian stimulation parameters of each group are listed in Table 2.

**Table 2:** Ovarian stimulation parameters.

**Group**	**1**	**2**	**3**	**4**	**5**	**6**	***P*-value**
N	879	482	858	1306	664	13708	
**OS protocol**							<0.001
GnRH agonist	494 (56.4%)	198 (41.1%)	356 (41.7%)	428 (33.1%)	555 (84.1%)	12321 (90.1%)	
GnRH antagonist	293 (33.4%)	189 (39.2%)	274 (32.1%)	409 (31.6%)	96 (14.5%)	1215 (8.9%)	
Other	89 (10.2%)	95 (19.7%)	223 (26.1%)	456 (35.3%)	9 (1.4%)	139 (1.0%)	
**Gn type**							<0.001
Recombinant-FSH	325 (37.9%)	104 (22.3%)	154 (18.8%)	83 (7.1%)	343 (52.3%)	8300 (60.7%)	
Urinary -FSH	533 (62.1%)	362 (77.7%)	664 (81.2%)	1090 (92.9%)	313 (47.7%)	5372 (39.3%)	
**FSH starting dose, IU**							<0.001
≤ 150	20 (4.4%)	7 (3.1%)	30 (6.5%)	34 (5.8%)	48 (13.7%)	1682 (18.9%)	
>150, ≤ 300	253 (56.0%)	40 (17.9%)	201 (43.3%)	110 (18.9%)	233 (66.6%)	5749 (64.6%)	
>300	179 (39.6%)	176 (78.9%)	233 (50.2%)	439 (75.3%)	69 (19.7%)	1469 (16.5%)	
Total Gn dose IU	2999.9 ± 1100.2	3060.8 ± 1184.4	2950.7 ± 1273.0	2919.3 ± 1357.4	2783.8 ± 1039.2	2356.3 ± 971.2	<0.001
Total Gn days	10.3 ± 2.8	9.5 ± 2.9	9.4 ± 3.5	8.5 ± 3.7	11.1 ± 2.8	10.4 ± 2.2	<0.001
HMG dose	1119.7 ± 1090.8	1213.8 ± 1112.8	1195.0 ± 1133.8	1306.1 ± 1162.5	1224.7 ± 1165.9	804.6 ± 845.7	<0.001

*FSH, follicle stimulating hormone; Gn, gonadotrophin; GnRH, gonadotrophin releasing hormone; HMG, human menopausal gonadotropin; OS, ovarian stimulation*.

*Mean + SD / N (%), calculated using EmpowerStats (www.empowerstats.com) and R*.

*Kruskal Wallis Rank Test for continuous variables, Chi-square testsfor categorical variables, Fisher Exact for categorical variables with Expects <10*.

### Embryo Transfer Policy

The oocyte processing and embryo development procedures as well as the embryo scoring system were described in our previous articles (8, 9). Grade 1–3 embryos on day 3 were considered useable embryos, and Grade 1–2 embryos were considered good-quality embryos. All fresh embryo transfers (ETs) were carried out on day 3 or day 5. In cases with sufficient number (≥ 3–4) of good-quality embryos on day 3, blastocyst transfer on day 5 would be practiced. Apart from the transferred embryos, patients' extra embryos were vitrified on day 3 or on blastocyst stage (day 5–6). Grade 1–3 cleavage stage embryos on day 3 and blastocysts with Gardner score above 4CC were cryopreserved (Cryo-top, open system, Kuwayama). The methods and Frozen ET procedure are detailed in previous verification study by our team (8, 9). If the implantation failed in fresh cycle, the frozen-thawed embryo transfer (FET) would be carried out using the remaining vitrified embryos or blastocysts. Patients under the age of 35 with good quality embryos were encouraged to receive a single-embryo transfer. A single embryo transfer policy was also applied for the patients who have the be abnormal uterus (e.g., scarred uterus, uterine malformation) and/ or other cases conflicted with twin pregnancy. Progesterone intramuscular injection (60 mg/day) was given for luteal phase support from the oocyte retrieval day until a negative serum beta-hCG or 8 weeks of pregnancy.

### Primary Outcome Measurements and Statistical Analysis

The primary outcome was cumulative live birth (CLB) defined as at least one live birth resulting from one aspirated ART cycle in the fresh ET or in the subsequent FET in relation to the number of oocytes retrieved. The numerator of CLBR calculation was the sum of live births achieved in the FETs and live births in fresh cycles. Only the first delivery was counted in the analysis if a patient achieved multiple deliveries. The CLBR was defined the cumulative live birth per transvaginal oocyte aspiration accordant to terminology definition (7, 10) One treatment cycle was defined as an oocyte retrieval. One complete treatment cycle referred to a treatment cycle that reached live birth or a treatment cycle that failed to reach live birth with all the embryos transferred. The cumulative live birth rate in this study was calculated based on the complete treatment cycle, so the patients (*n* = 337) of non-complete treatment were excluded.

The data processing and statistical analysis were performed using EmpowerStats software (www.empowerstats.com) and statistical software packages R. To assess the odds ratio (OR) of CLBR in different patient groups, a multiple variables regression model was established with potential confounding factors as the variables and adjusted for the year of treatment, female BMI, type of infertility, length of infertility, gravidity, parity, main etiology, OS protocol, gonadotrophin type, and FSH starting dose. Patients were enrolled for 3 years, during which IVF procedure was revised. To eliminate bias caused by this factor, the cumulative live birth rate was adjusted for the year of treatment. Female BMI, type of infertility, length of infertility, gravidity, parity, and main etiology were important factors affecting pregnancy and live birth through experience or literature. OS protocol, gonadotrophin type and FSH starting dose are the key indicators to affect the number of oocytes retrieved and ultimately the cumulative chance of live births.

## Results

### Oocyte and Embryo Parameters

As shown in Table 3, the number of oocytes retrieved decreased in low prognosis patients from group 1 to group 4 (*p* < 0.001), as well as number of 2 pro-nucleus (2PN) zygotes (*p* < 0.001), number of day 3 usable embryos (*p* < 0.001) and number of good quality embryos (*p* < 0.001). Oocyte output rate (number of oocyte retrieved/AFC x 100%) was highest in group 3 (145.3%), followed by group 4 (101.2%), group 1 (74.0%), and group 2 (67.3%).

**Table 3:** Oocytes and embryo parameters and CLBRs.

**Group**	**1**	**2**	**3**	**4**	**5**	**6**	***P*-value**
N	879	482	858	1306	664	13708	
Number of Oocytes/AFC	74.00%	67.30%	145.30%	101.20%	90.50%	91.40%	<0.001
Cycles of 0 oocyte retrieved (%)	14 (1.6%)	20 (4.1%)	56 (6.5%)	157 (12.0%)	1 (0.2%)	60 (0.4%)	<0.001
Number of oocytes	7.4 ± 4.8	5.1 ± 3.7	4.3 ± 3.7	2.9 ± 2.9	12.6 ± 6.2	12.3 ± 6.7	<0.001
Number of 2PN	4.4 ± 3.3	3.3 ± 2.5	2.8 ± 2.5	2.0 ± 2.0	7.0 ± 4.3	7.4 ± 4.6	<0.001
Number of day 3 usable embryos	3.4 ± 2.8	2.6 ± 2.2	2.3 ± 2.2	1.6 ± 1.7	5.0 ± 3.7	6.2 ± 4.2	<0.001
Number of day 3 good quality embryos	1.8 ± 2.1	1.5 ± 1.8	1.3 ± 1.7	0.9 ± 1.3	2.5 ± 2.7	3.7 ± 3.3	<0.001
Cumulative live births (rate %)	392 (44.6%)	118 (24.5%)	305 (35.5%)	166 (12.7%)	355 (53.5%)	9164 (66.9%)	<0.001

*AFC, antral follicle count; PN, pronucleus*.

*Mean + SD / N (%), calculated using EmpowerStats (www.empowerstats.com) and R*.

*Kruskal Wallis Rank Test for continuous variables, Chi-square tests for categorical variables, Fisher Exact for categorical variables with Expects <10*.

### Pregnancy Outcomes and Cumulative Live Birth Rate (CLBR)

Inconsistent with the distribution pattern of number of oocytes and embryos by group, the CLBRs in the order from highest to lowest was 44.6% in group 1(*n* = 879), 35.5% in group 3(*n* = 858), 24.5% in group 2 (*n* = 482) and 12.7% in group 4 (*n* = 1306). A multiple-variable regression analysis was performed with variables that may act as confounding factors described in Tables 1, 2. The adjusted odds ratios (ORs) of CLBR with their 95% confidence intervals (CIs) were shown in Table 4. Consistent with the trend of non-adjusted results, the CLBR in group 3 (OR 0.9, 95% CI 0.7–1.2, *p* = 0.615) was slightly lower and group 5 (OR 1.3, 95% CI 0.9–1.7, *p* = 0.111) was slightly higher than CLBR in group 1 without significant statistical difference. The CLBR in group 2 was significantly lower than in group 1 (OR 0.6, 95% CI 0.4–0.8, *p* = 0.004) and CLBR in group 4 was the lowest (OR 0.4, 95% CI 0.3–0.6, *p* < 0.001) as compared to group 1. Table 5 showed the pregnancy outcomes per fresh transfer or FET in low prognosis patients. The implantation rates in aged groups (group 2 and group 4) were significantly lower than in young groups (group 1 and group 3).

**Table 4:** Logistic regression analysis for CLBRs.

**Group**	**Non-adjusted OR (95% CI), *P*-value**	**Adjusted OR (95% CI), *P*-value**
1	1	1
2	0.4 (0.3, 0.5), *p* < 0.001	0.6 (0.4, 0.8), *p* = 0.004
3	0.7 (0.6, 0.8), *p* < 0.001	0.9 (0.7, 1.2), *p* = 0.615
4	0.2 (0.1, 0.2), *p* < 0.001	0.4 (0.3, 0.6), *p* < 0.001
5	1.4 (1.2, 1.7), *p* < 0.001	1.3 (0.9, 1.7), *p* = 0.111
6	2.5 (2.2, 2.9), *p* < 0.001	2.0 (1.6, 2.4), *p* < 0.001

*OR, odds ratio. OR was adjusted for the year of treatment, female BMI, type of infertility, length of infertility, gravidity, parity, main etiology, OS protocol, Gn type and FSH starting dose*.

**Table 5:** Pregnancy outcomes per transfer both fresh and frozen embryo transfer in low prognosis patients.

**Group**	**1**	**2**	**3**	**4**	**5**	**6**	***P*-value**
Transfer cycle (fresh ET + FET)	1,126	602	781	976	1,199	18,862	
Number of embryos transferred	1.8 ± 0.5	1.9 ± 0.5	1.7 ± 0.5	1.7 ± 0.6	1.9 ± 0.5	1.7 ± 0.5	<0.001
Implantation rate	34.40%	21.26%	40.45%	19.19%	29.49%	48.24%	<0.001
Pregnancy loss rate/ transfer	9.68%	10.80%	9.99%	9.22%	9.84%	9.47%	0.896
Miscarriage	7.46%	9.14%	6.91%	8.20%	6.84%	6.56%	0.059
in first trimester/ transfer							

*ET, embryo transfer; FET, Frozen embryo transfer*.

*Kruskal Wallis Rank Test for continuous variables, Chi-square tests for categorical variables, Fisher Exact for categorical variables with Expects <10*.

**Figure 2:**
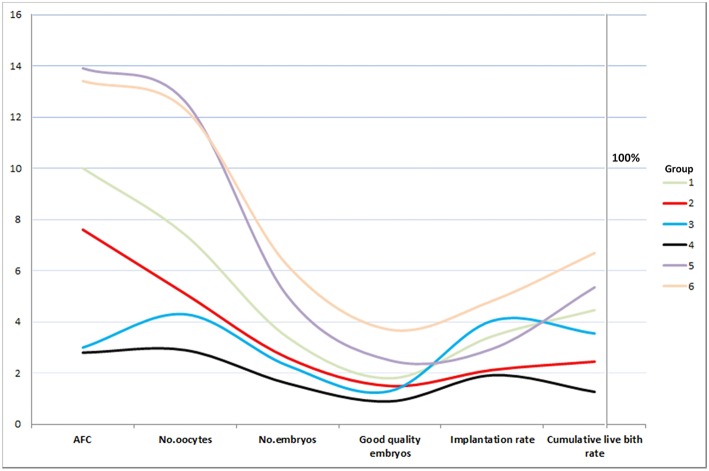
Trend chart of key events in low prognosis patients. There was a crossing of trend lines between group 2 (red) and group 3 (blue) after embryo transfer. X axis represents the average number of AFC, number of oocytes, number embryos, number of good quality embryos, rate of implantation and rate of cumulative live birth. The Y axis on the left represents the number of the first four variables (*n*) and the Y axis on the right represents the rate of last two variables (%).

**Figure 3:**
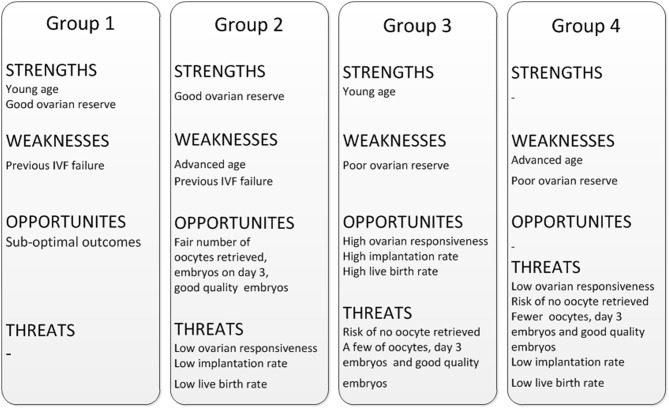
SWOT analysis of 4 groups of low prognosis patients defined by POSEIDON criteria.

Figure 2 showed the trend chart of key events in low prognosis patients. There was a crossing of trend lines between group 2 and group 3 after embryo transfer. Patients in group 2 (age ≥35; AFC ≥ 5) had higher AFC, more oocytes retrieved, more embryos and more good quality embryos, but decreased implantation rate and CLBR. On the contrary, though patients in group 3 (age < 35; AFC < 5) had fewer oocytes and embryos, the CLBR turned out higher than that in group 2. The SWOT analysis of 4 groups of low prognosis patients defined by POSEIDON criteria is shown in Figure 3.

## Discussion

The main finding of this retrospective study in POSEIDON criteria-defined population was that the CLBR was highest in group 1, followed by group 3 and group 2, and lowest in group 4. According to our results, the CLBR from the young women with poor ovarian reserve (group 3) was slightly lower than that from young women with good ovarian reserve and previous low responder (group 1). Though the patients in group 2 (age ≥ 35; AFC ≥ 5) had better ovarian reserve, more oocytes and more embryos, the CLBR and implantation rate, on the other way round, were lower than in group 3 patients with poorer ovarian reserve, fewer oocytes and fewer embryos. This finding may facilitate the development of management strategies for low prognosis patients.

The innovative POSEIDON criteria aim at identifying and stratifying low prognosis patients into four distinct groups based on female age, AFC and ovarian response in the previous cycle (4). The patients in group 2 were characterized by good ovarian reserve and advanced age, exactly the opposite of group 3 patients with poor ovarian reserve but are at younger age. Studies (11–13) have shown that CLBR increases with the number of oocytes retrieved even in the women of advanced age (14). It was suggested the number of oocytes retrieved is a very important variable independently associated with CLBR. Patients in group 2 with a higher number of oocytes were expected to have a better prognosis than patients in group 3, because group 2 patients had more embryos to transfer. However, the CLBR and implantation rate were reversely higher in group 2 than in group 3.

Our results are consistent with previous studies (15, 16) on the association of ovarian reserve and pregnancy outcome. Chang et al. (15) found that there were lower rates of normal fertilization, cleavage, high-quality embryos, implantation, and pregnancy in older women than in younger women with diminished ovarian reserve. The primary reason was the adverse impact of aging oocyte on the pregnancy outcome (low implantation rate and high pregnancy loss rate) due to chromosomal abnormalities (17) and cytoplasmic dysfunction (18). The decline in fertility with aging involves both quantity and quality of oocyte. Implantation and miscarriage are related to the quality of oocytes but not necessarily the ovarian reserve (16). The fair ovarian reserve in group 2 would increase the possibility of obtaining more oocytes and embryos to transfer, but at the same time, the higher aneuploidy rate would lead to low implantation rate and high miscarriage rate.

In terms of the management of patients in group 2, more attention should be paid to develop strategies of improving the oocyte quality rather than oocyte quantity or embryo quantity. Because more embryos are achieved on day 3 in group 2, culturing embryos to blastocyst stage for transfer is a good option. Day 5–6 embryos have lower rate of segmental aneuploidy (19) and higher viability for implantation (20) than day 2–3 embryos. Preimplantation genetic testing for aneuploidies (PGT-A) are also beneficial for advanced women to select an euploid embryo to transfer (21). An optimal ovarian stimulation regimen to improve the quality of oocytes (22, 23) could be an alternative option. Supplements such as dehydroepiandrosterone were tried to improve follicle development (24), though there is insufficient evidence to support their use in these patients.

The patients in group 3 (age < 35; AFC < 5) had a poor ovarian reserve, who were expected poor responders with poor pregnancy outcome. Interestingly, the oocytes output rate (145.3% oocytes retrieved per AFC) in group 3 was significantly higher than in the other groups, though FSH starting dose was not significantly increased (Table 2). This suggested the response of antral follicles to gonadotropin may have reached the limit of its ability, therefore there will be no additional benefit in oocyte number to further increase daily gonadotrophin doses (25). Evaluating ovarian sensitivity to FSH is a key element to improve IVF success rates in these low prognosis patients and open new treatment perspectives (26). The high oocytes output rate per AFC in group 3 also supported the reported recommendation of maximum daily dose of 300 IU rFSH (5). For the patients in group 3, more efforts should be focused on increasing the number of oocytes, as the clinical pregnancy outcome is reassured once oocytes are acquired.

The patients in group 5 were the non-low prognosis patients who had good ovarian response (more than 9 oocytes in previous retrieval), however most of these patients failed to live birth in previous IVF cycle. Comparing with another non-low prognosis patients with first IVF treatment (group 6), though the patients in group 5 have more AFC and number of oocytes retrieved, the embryo development and pregnancy outcome were inferior to that in group 6. Therefore, the failure in first IVF patients with good ovarian response may be the poor prognosis for subsequently IVF treatment.

Pelvic-tubal factor is the most common cause of infertility, accounting for about 10.8–78.3% of infertile women in China (27, 28). Tubal factor mainly involves tubal occlusion and peritoneal pathology causing adhesions, which was diagnosed by hysterosalpingography and laparoscopy. The prevalent cause of tubal factor infertility was attributed to pelvic inflammatory disease (PID), salpingitis and endometriosis (29). In the patient groups of this study, tubal factor is the main infertility etiology, ranging from 62.9 to 71.4%, which was higher than that in infertility women of other countries and regions. Therefore, it should be careful to interpret the wider implications of the findings.

Limitations are related to the retrospective nature of the study and the fact that the data was from a single center also weakens the universality of our observations. Other potential limitations could be that non-GnRH analog protocol was used in some women with diminished ovarian reserve rather than in those with normal ovarian reserve. The fresh cycles that failed to yield any oocyte were not included in this study, for example, cycles with cancellation of ovarian stimulation.

The results of this study may provide new insights for the development of management strategies for low prognosis patients. A SWOT analysis was performed to help the management for poor prognosis patients in clinic, which was drawn from the POSEIDON reports (1, 2, 4, 5) and the data in this study. The responsiveness of antral follicles to gonadotrophin was extremely higher in group 3 than in the other groups. Considering the gratifying CLBR outcome in group 3, in order to increase oocyte yield, we suggest to try more ovarian stimulations but not harder ovarian stimulation through excessive daily gonadotrophin dose. Though the patients in group 2 have more ovarian reserve as well as more oocytes and embryos, the CLBR was lower than expected. The management strategy for group 2 should be improving the live birth rate rather than increasing number of oocytes retrieved.

## Conclusion

In conclusion, inconsistent with the distribution pattern of oocyte quantity and embryo quantity by patient group, the CLBRs in the order from highest to lowest were in group 1 (young women with good ovarian reserve), group 3 (young women with poor ovarian reserve), group 2 (women at advanced age with good ovarian reserve), and at last group 4 (women at advanced age with poor ovarian reserve.

## Data Availability

The raw data supporting the conclusions of this manuscript will be made available by the authors, without undue reservation, to any qualified researcher.

## Ethics Statement

The study was approved by the Ethics Committee for the Clinical Application of Human Assisted Reproductive Technology of Northwest Women's and Children's Hospital (No. 2018002). The ethics committee that approved this study waived the need to obtain informed consent. All of the research was performed in accordance with the relevant guidelines and regulations.

## Author Contributions

WS and JS conceived and designed the study. WS and WZ drafted and revised the manuscript. WS, ZZ, and WZ analyzed and interpreted the data. LT, ZZ, and HZ collected and cleared the data. All authors have read and approved the final version of the manuscript.

### Conflict of Interest Statement

The authors declare that the research was conducted in the absence of any commercial or financial relationships that could be construed as a potential conflict of interest.
